# Association of HLA-DRB1*11 and HLA-DRB1*12 gene polymorphism with COVID-19 in Burkina Faso

**DOI:** 10.1186/s12920-023-01684-8

**Published:** 2023-10-16

**Authors:** Alfred Rakissida Ouedraogo, Lassina Traoré, Abdoul Karim Ouattara, Alexis Rakiswende Ouedraogo, Sidnooma Véronique Zongo, Mousso Savadogo, Tatiana Doriane Lallogo, Herman Karim Sombie, Pegdwendé Abel Sorgho, Teega-wendé Clarisse Ouedraogo, Florencia Wendkuuni Djigma, Assita Sanou Lamien, Albert Théophane Yonli, Olga Mélanie Lompo, Jacques Simporé

**Affiliations:** 1 Laboratoire de Morphologie et Organogénèse (LAMO), Université Joseph KI-ZERBO, UFR/SDS, Ouagadougou 01, 01 BP 7021 Burkina Faso; 2 Université Joseph KI-ZERBO, Laboratoire de Biologie Moléculaire et de Génétique (LABIOGENE), UFR/SVT, Ouagadougou 01, 01 BP 7021 Burkina Faso; 3 Université Norbert ZONGO - Centre Universitaire de Manga, BP 376, Koudougou, Burkina Faso; 4 Centre de Recherche Biomoléculaire Pietro Annigoni (CERBA), P.O. Box 364, Ouagadougou 01, Burkina Faso

**Keywords:** Severe COVID-19, HLA-DRB1*11, HLA-DRB1*12, Genotypes, Burkina Faso

## Abstract

**Background:**

The clinical manifestations of coronavirus disease (COVID-19) can vary widely, ranging from asymptomatic to severe, and may be influenced by the host genetic background. The aim of the present study was to determine the frequencies of HLA-DRB1*11 and HLA-DRB1*12 allele polymorphisms and their associations with COVID-19.

**Methods:**

In this cross-sectional study, 198 subjects were enrolled, including 150 COVID-19 positive cases and 48 subjects who tested negative for COVID-19. Participants were recruited from the emergency, intensive care, and infectious diseases departments of the Bogodogo Centre University Hospital (CHU-B) or the routine laboratory of Centre de Recherche Biomoléculaire Pietro Annigoni (CERBA). Genomic DNA was extracted from nasopharyngeal swabs samples and multiplex PCR-SSP was used to detect the HLA-DRB1*11 and HLA-DRB1*12 alleles. The study was approved by CERS (№ 2021-02-033).

**Results:**

The positive cases were categorized into 38 asymptomatic (CC+), 60 symptomatic (NC+), and 52 severe cases (SC+). Females were more frequent in the overall study population (53.0%, 105/198) as well as in the negative group’s CC- (68.75%, 33/48) and SC+ (57.69%, 30/52 negative groups, whereas males were more frequent in the CC+ (63.16%, 24/38) and NC+ (53.33%, 32/60) groups. The highest mean age was observed in the SC + group. A frequency of 19.19% (38/198) and 14.65% (29/198) was found for the HLA-DRB1*11 and HLA-DRB1*12 alleles, respectively. Individuals carrying the HLA-DRB1*11 allele had an approximately sixfold higher risk of asymptomatic SARS-CoV-2 infection (OR = 5.72 [1.683–19.442], p = 0.005) based on the association analysis.

**Conclusions:**

Altogether, the present study reports high frequency of HLA-DRB1*11 and HLA-DRB1*12 alleles within a population from Ouagadougou, Burkina Faso. The results suggest that individuals carrying the HLA-DRB1*11 allele are more susceptible to COVID-19 infection but may not display symptoms.

**Supplementary Information:**

The online version contains supplementary material available at 10.1186/s12920-023-01684-8.

## Background

In December 2019, a group of pneumonia patients in Wuhan, China, were discovered to have a new disease now known as COVID-19, caused by the severe acute respiratory syndrome coronavirus 2 (SARS-CoV-2) [1]. This disease was declared a global pandemic on March 11th, 2020, by the World Health Organization (WHO) [2]. From the outset, the pandemic has impacted the world in several ways with devastating consequences on public health, resulting in a significant loss of life across the world, particularly among vulnerable populations. According to recent estimates from the World Health Organization (WHO), as of September 6, 2023, there have been 770,437,327 confirmed cases of COVID-19 reported worldwide, with 6,956,900 recorded deaths [3]. In addition, the pandemic has led to the closure of various activities with significant financial losses, widespread unemployment, and a sharp decline in productivity. Africa was originally predicted to be the hardest hit continent due to limited resources and fragile health systems. Surprisingly, however, the continent was the least affected, although the reasons for this remain unclear.

Research has been conducted to understand why the African continent was less affected by SARS-CoV-2. The literature suggests that certain human host genes, such as CCR5, DC-SIGN, APOBEC, HLA, ACE, GSTM, GSTT, KIR, etc., may influence the onset and progression of viral infections [4–7]. In addition, polymorphisms in genes including ACE-2, TMPRSS2, vitamin D receptor, vitamin D binding protein, CD147, 78 kDa glucose-regulated protein, dipeptidyl peptidase-4 (DPP4), neuropilin-1, heme oxygenase, apolipoprotein L1, vitamin K epoxide reductase complex 1 (VKORC1) have been associated with the modulation of asymptomatic, symptomatic and severe forms of COVID-19 [8].

A genome-wide study has associated specific regions and polymorphisms of immunoregulatory genes, such as those within the HLA system, with the severity of COVID-19 [9]. The HLA system, located on the short arm of chromosome 6, is a complex of more than 200 genes, including more than 40 that encode leukocyte antigens [10]. The HLA genes involved in the immune response are divided into three classes, I, II, and III, and play a critical role in regulating the immune response to foreign antigens and in distinguishing between self and non-self [11]. Previous studies have shown that individuals with severe COVID-19 have lower expression of HLA-DR [12]. In addition, individuals with HLA-DRB1*11 and HLA-DRB1*15 were found to have a reduced risk of developing severe forms of coronavirus disease [13]. While numerous studies have identified specific HLA alleles that confer protection against SARS-CoV-2 infection, many of these studies were conducted in areas with high rates of COVID-19 and significant mortality. Therefore, it remains unclear whether these findings extend to regions such as sub-Saharan Africa. To address this knowledge gap, the present study aimed to investigate whether HLA-DRB1*11 and HLA-DRB1*12 polymorphisms are associated with COVID-19 pathogenesis in Burkina Faso.

## Results

### Socio-demographic characteristics of the study population and COVID-19 status

The study included a population of 198 individuals categorized into four groups, including 48 (24.2%) COVID-19 negative cases (CC-) and 150 positive cases, including 38 (19.2%) asymptomatic positive cases (CC+), 60 (30.3%) symptomatic positive cases (NC+), and 52 (26.3%) severe positive cases (SC+).

Females represented the majority of the study population (53.0%, 105/198), including the CC- (68.75%, 33/48) and SC+ (57.69%, 30/52) groups, whereas males predominated in the CC+ (63.16%, 24/38) and NC+ (53.33%, 32/60) groups. The mean age of controls was 24.60 ± 17.91 years, while the mean age of cases was 30.21 ± 11.41 years for CC+, 45.72 ± 21.78 years for NC+, and 57.06 ± 16.57 years for SC+. The most represented age groups were 16–35 years (63.2% of CC+) and 36–64 years (46.2% of SC+), according to data categorization by age (Table 1).

**Table 1:** Sociodemographic characteristics of the study population and COVID-19 status

Variables		N	Percentage (%)
**Status**	CC-	48	24.2
	CC+.	38	19.2
	NC+.	60	30.3
	SC+.	52	25.3
**Gender**	F	93	53.0
	H	105	47.0
**Age group**	<5 years	6	3.0
	6 to 15 years	12	6.1
	16 to 35 years old	74	37.4
	36 à 64	68	34.3
	≥ 65 years	38	19.2
**Tota**l		198	100

***Legend***: *CC-: Negative cases; CC+: asymptomatic positive case; NC+: symptomatic positive case; SC+: severe positive cases*

### Allelic and genotypic frequencies of ***HLA-DRB1*** in the study population

The HLA-DRB1*11 and HLA-DRB1*12 alleles were identified in the study population by PCR-SSP (supplementary file 1). The frequency of the HLA-DRB11*1 allele was 19.19% (38/198), and the frequency of the HLA-DRB1*12 allele was 14.65% (29/198). Within the different groups, the frequency of the HLA-DRB1*11 allele ranged from 8.33% (4/48) in the CC- group to 34.21% (13/38) in the CC + group, 20% (12/60) in the NC + group, and 17.31% (9/52) in the SC + group. The HLA-DRB1*12 allele frequency ranged from 6.25% (3/48) in the CC- group to 21.05% (8/38) in the CC + group, 17.31% (9/52) in the SC + group, and 15.00% (9/60) in the NC + group (Table 2). The HLA-DRB1*11/12 genotype was found in 6.1% (12/198) of the individuals in the present study, with the highest frequency (11.54%; 6/52) in the SC + group (Table 3). The heterozygotes represented 21.72% (43/198) of the study population.

**Table 2:** Distribution of HLA-DRB1 alleles in the study population

Clinical status	HLA-DRB1*11				HLA-DRB1*12			
	n (%)	p-value	OR	95% CI	n (%)	p-value	OR	95% CI
CC- (n = 48) *	4 (8.33)				3 (6.25)			
CC+ (n = 38)	13 (34.21)	**0.005**	**5.72**	**[1.683–19.442]**	8 (21.05)	0.054	4.00	[0.981–16.302]
SC+ (n = 52)	9 (17.31)	0.240	2.302	[0.659–8.040]	9 (17.31)	0.089	3.140	[0.796–12.378]
NC+ (n = 60)	12 (20.00)	0.108	2.75	[0.826–9.160]	9 (15.00)	0.150	2.65	[0.675–10.383]
Total (n = 198)	38 (19.19)				29 (14.65)			

***Legend***: *: Group compared with other groups with significant p-value in bold;***Legend***: *CC-: Negative cases; CC+: asymptomatic positive case; NC+: symptomatic positive case; SC+: severe positive cases*

**Table 3:** Distribution of HLA-DRB1*11/12 genotypes in the study population

Clinical status	Genotypes HLA-DRB1*11/12						
	+/+	+/-	-/+	-/-	p-value	OR	95% CI
CC- (n = 48)	2 (4.2)	2 (4.2)	1 (2.1)	43 (89.6)	0.651^a^	1.971	[0.312–12.444]
CC+ (n = 38)	3 (7.9)	10 (26.3)	5 (13.2)	20 (52.6)	0.725^b^	1.533	[0.307–7.665]
SC+ (n = 52)	6 (11.5)	3 (5.8)	3 (5.8)	40 (76.9)	0.728^c^	1.522	[0.356–6.513]
NC+ (n = 60)	1 (1.7)	11 (18.3)	8 (13.3)	40 (66.7)	0.296^d^	0.198	[0.020–1.975]
Total (n = 198)	12 (6.1)	26 (13.1)	17 (8.6)	143 (72.2)	**0.048** ^**e**^	**7.695**	**[0.895–66.189]**

***Legend***: *CC-: Negative cases; CC+: asymptomatic positive case; NC+: symptomatic positive case; SC+: severe positive cases.* Comparison for HLA-DRB1*11/12 genotype **+/+**^a^: CC- vs. CC+; ^b^: CC- vs. (SC+ & NC+); ^c^: CC + vs. SC+; ^d^: CC + vs. NC+; ^e^: SC + vs. NC+.

### Correlation between ***HLA-DRB1*** alleles and genotypes and SARS-CoV-2 infection

Table 2 shows a comparison of the allelic profiles between positive cases (CC+, NC + and SC+) and negative cases (CC-). The frequency of the HLA-DRB1*11 allele was significantly higher in CC + cases (34.21%, 13/38) than in CC- (8.33%, 4/48) with p = 0.005 and OR = 5.72 [1.683–19.442], whereas no significant difference was found in the HLA-DRB1*12 allele (p = 0.054).

There was no statistical difference between negative CC- cases and NC + and SC + cases or between CC + cases and NC + and SC + cases for both HLA-DRB1*11 and HLA-DRB1*12 alleles (p > 0.05). However, the HLA-DRB1*11/12 genotype was significantly higher in SC + cases (11.54%, 6/52) than in the NC + group (1.67%, 1/60) with p = 0.048 and OR = 7.70 [0.895–66.189].

## Discussion

The worldwide impact of the COVID-19 pandemic has been significant, with an estimated 770,437,327 confirmed cases and 6,956,900 deaths as of September 6, 2023 [3]. The disease progression and susceptibility to SARS-CoV-2 are influenced by both environmental and genetic factors [14], and several genetic and epidemiologic studies have suggested the involvement of HLA system genes in COVID-19 development [9]. In this cross-sectional study, we investigated the potential role of HLA-DRB1*11 and HLA-DRB1*12 alleles in individuals tested for SARS-CoV-2 infection.

Although studies have reported male sex as a major risk factor for the severity of COVID-19 in Asian [15] and European [16] populations, the present study found a higher proportion of females (57.69%, 30/52) in the SC + group. This difference may be due to the fact that biological sex affects the aging of the immune system, with women having stronger immunity to viral infections [17]. However, comorbidities may have played a role in the higher proportion of severe cases among women in the present study. In addition, it should be noted that the rate of SARS-CoV-2 infection also varies with age [17] and that male predominated in the CC + and NC + groups in the present study.

The severity of COVID-19 is known to increase with age, with patients older than 60 years having the highest percentage of patients with poor prognosis and admission to intensive care [18]. In the present study, the age groups 36–64 years (24/52) and over 64 years (21/52) accounted for 86.54% (45/52) of SC + patients, with the highest mean age (57.06 years). Several studies have shown that the immune system weakens with age, exposing the elderly to a more severe form of disease caused by coronaviruses [19, 20].

The elderly population is often affected by comorbidities such as hypertension, cardiovascular disease, and diabetes, which can increase the severity of COVID-19 even in younger individuals [18]. The present study focused on individuals who had been exposed to SARS-CoV-2 infected individuals. Analysis of two *HLA-DRB1* alleles revealed that HLA-DRB1*11 was more common, with a frequency of 19.19%, compared with HLA-DRB1*12, which had a frequency of 14.65%. These findings are consistent with data from the Allele Frequency Net Database (AFND), which shows a higher prevalence of HLA-DRB1*11 (17%) compared to HLA-DRB1*12 (1%) in the Mossi ethnic group, the largest ethnic group in Burkina Faso [21]. However, other studies by Zouré et *al.* [22] and Lallogo et al. [23] reported a higher frequency of HLA-DRB1*12 than HLA-DRB1*11 in individuals at risk for breast cancer and HIV serodiscordant couples in Burkina Faso, respectively. This may be due to differences in the study populations. Previous studies have suggested that HLA-DRB1*11 may provide protection against severe forms of COVID-19 [13] and since the present study mainly included non-severe cases, it is possible that the prevalence of HLA-DRB1*11 was higher in this population.

SARS-CoV-2 has affected countries worldwide with varying rates of infection, ranging from a few cases to thousands reported daily (20). The outcomes of infection have also varied, ranging from asymptomatic cases to severe complications and even death. Studies have shown that individuals of African American descent are more susceptible to COVID-19 complications, highlighting the role of the immune system in responding to the virus [24]. To investigate susceptibility to infection, studies have examined the association of certain HLA polymorphisms with disease [13, 24–29]. The present study investigated the association between HLA-DRB1*11 and HLA-DRB1*12 alleles and the risk of SARS-CoV-2 infection. The results of the present study suggest that among individuals who had been in contact with infected individuals but remained asymptomatic, those carrying the HLA-DRB1*11 allele had approximately a 6-fold higher risk of testing positive for SARS-CoV-2 (p = 0.005; OR = 5.72 [1.683–19.442]). Therefore, the HLA-DRB1*11 allele would not provide protection against infection but rather against the development of symptoms within our study population. This is consistent with a meta-analysis by Zorana et *al.* [30], showing that the HLA-DRB1*11 allele is associated with a reduced risk of severe symptoms requiring hospitalization or intensive care. HLA-DRB1*11 was also found to be independently associated with COVID-19(+) and a 40% decrease in the probability of COVID-19(+) in kidney transplant recipients [31]. In a recent case reports study, HLA-DRB1*11:01 was speculated to be involved in the pathogenesis of Graves’ disease following SARS-CoV-2 vaccination [32]. However, patients with HLA-DRB1*11/12 genotypes had an approximately 8-fold risk of developing severe forms of COVID-19 (p = 0.048; OR = 7.70 [0.895–66.189]). In a study conducted in Japan, HLA-DRB1*12:01 and DRB1*12:02 were exclusively found in direct antiglobulin test (DAT)-positive COVID-19 patients [26]. Polymorphisms in the HLA system contribute to the selection of antigenic peptides for presentation to T cells, resulting in different immune responses among individuals. Due to the large diversity of haplotype combinations in the HLA system, further investigation of other *HLA-DRB1* alleles is needed to confirm these findings.

While there is no conclusive evidence of a clear association between haplotypes containing the HLA-DRB1*12 allele and susceptibility to coronavirus disease, several studies have indicated that it is comparatively less prevalent in infected populations compared to control groups [26, 33]. It is noteworthy that the small sample size is one of the limitations of our study. Additionally, only two HLA system polymorphisms were investigated. However, the study provides a significant insight into HLA-DRB1*11 and HLA-DRB1*12 alleles in the context of SARS-CoV-2 infection in Burkina Faso.

## Conclusion

The present study reports a high frequency of the HLA-DRB1*11 allele compared to HLA-DRB1*12 in the study population. The results suggest that carrying the HLA-DRB1*11 allele increases the risk of asymptomatic SARS-CoV-2 infection. Although the sample size may limit the ability to draw definitive conclusions about the various allelic combinations of the HLA class II system, further analysis may provide interesting insights.

## Materials and methods

### Study population

This was a cross-sectional study of 198 individuals, all recruited from either the Emergency, Intensive Care and Infectious Diseases Departments of CHU de Bogodogo or the routine laboratory of CERBA. The study population was divided into four different groups based on clinical and biological status, namely negative cases (CC-), asymptomatic positive cases (CC+), symptomatic positive cases (NC+), and severe positive cases (SC+).

The CC- subgroup consisted of individuals who tested negative for COVID-19 by PCR, while those without clinical symptoms who tested positive were included in the CC + subgroup. The NC + subgroup consisted of individuals with symptoms such as fever, cough and difficulty breathing who tested positive for SARS-CoV-2 nucleic acids by PCR. The SC + subgroup consisted of symptomatic patients positive for SARS-CoV-2 nucleic acids by PCR with at least one of the severity factors, such as increased respiratory rate (> 30/min), drop in blood pressure (SBP < 90 mmHg AND/OR DBP < 60 mmHg), impaired consciousness, state of shock, require oxygen supplementation at a rate exceeding 5 L/min to maintain arterial oxygen saturation (SpO_2_) between 90% and 95%, cardiac rhythm disturbances with hemodynamic consequences, according to the COVID-19 case definition in Burkina Faso. All patients with COVID-19 severe cases were hospitalized in the intensive care unit (ICU).

### Sampling

The samples were collected randomly during the period August 2020-August 2021 to form the four different groups. Collection of SARS-CoV-2 infected respiratory epithelial cells was performed by nasopharyngeal swabbing. Participants were seated in an examination chair with a headrest or on an examination bed in a semi-seated position with their head supported avoiding triggering the head-back reflex when inserting the swab. After obtaining the sample, the swab was placed in a tube and the distal part was broken off. The tube was then carefully resealed and decontaminated with virucide before being placed in a transport bag.

### DNA extraction

Genomic DNA was extracted from nasopharyngeal swabs of individuals using the KingFisher automated extractor (Thermo Fisher Scientific, Waltham, WA, USA) and the MagMax kit according to the manufacturer’s protocol. The extracted DNA was then stored at -20 °C until used for PCR amplification of the target alleles.

### PCR-SSP amplification

To detect the presence of HLA-DRB1*11 and HLA-DRB1*12 alleles, PCR-SSP was performed using the sequence-specific primers (Table 4) originally described by Ma et *al.* [31] with a slight modification. Due to the large number and variability of HLA alleles, the amplification reaction included a primer pair designed to target the human growth factor (HGF) housekeeping gene. This served as a control to verify the PCR in the event that the sample did not contain any of the alleles of interest.

**Table 4:** Sequence-specific primers for *HLA-DRB1* alleles

	Primer sequences		Size (bp)
Alleles	**5’-sequence**	**3’-sequence**	
DRB1*11	5’GTTTCTTGGAGTACTCTACGTC3’	5’CTGGCTGTTCCAGTACTCCT3’	176
DRB1*12	5’ACTCTACGGGTGAGTGTT3’	5’ACTGTGAAGCTCTCCACAG3’	244
*HGF*	5’CAGTGCCTTCCCAACCATTCCCTTA3’	5’ATCCACTCACGGATTTCTGTTGTGTTTC3’	432

The PCR of a sample was considered valid only if an amplification band for the HGF gene was observed. If the HGF gene amplification control band (432 bp) was not present, the PCR result for that sample was considered invalid. The presence of 176 and 244 bp bands indicated amplification of the HLA-DRB1*11 and HLA-DRB1*12 alleles, respectively. A multiplex PCR was performed using the GeneAmp PCR System 9700 (Applied Biosystem, Foster City, USA) to simultaneously target both alleles and the internal control, the HGF gene. The total reaction volume of 25 µL consisted of 7 µL molecular biology water; 10 µL 2X mix (Emerald Amp GT PCR Master Mix); 0.5 µL of each primer pair at a concentration of 0.2 µM; and 5 µL of each DNA extract at 10 ng/µL.

### Electrophoresis of PCR products

PCR products were subjected to nondenaturing electrophoresis on a 2% agarose gel with 0.1% ethidium bromide in 1X Tris-borate-EDTA (TBE) buffer at 100 millivolts for 45 min. The GeneFlash gel documentation system (Syngene, Bio-Imaging, UK) was used to visualize samples under ultraviolet light, and fragment size was determined using a 100-bp DNA ladder.

### Statistical analysis

The collected data were entered into Microsoft Excel 2019 spreadsheets and subsequently analyzed using R version 4.0.2 and SPSS version 20. The frequencies of HLA-DRB1*11 and 12 alleles were calculated by direct counting, and comparisons were made between the different groups. Statistical significance was determined to use either the chi-squared test or Fisher’s exact test, as appropriate for small numbers. A p-value of less than 0.05 was considered statistically significant.

### Electronic supplementary material

Below is the link to the electronic supplementary material.

Supplementary Material 1

## Data Availability

The datasets used and analyzed during the current study are available from the corresponding author on a reasonable request.

## Abbreviations

ACE: Angiotensin Converting Enzyme
APOBEC: Apolipoprotein B mRNA Editing Catalytic Polypeptide-like
CC-: Negative Contact Cases
CC+: Positive Contact cases
CCR5: CC chemokine receptor 5
CERBA: Center for Biomolecular Research Pietro Annigoni
CHU: University Hospital Center
COVID-19: Coronavirus disease of 2019
DC-SIGN: Dendritic Cell-Specific ICAM-Grabbing Non-integrin
DRSC: Direction Régionale de la Santé du Centre
GSTM: Glutathione S-Transferase Mu
GSTT: Glutathione S-Transferase Theta
HGF: Human Growth Factor
HLA: Human Leukocyte Antigen
KIR: Killer immunoglobulin-like receptor
LABIOGENE: Laboratoire de Biologie Moléculaire et de Génétique
LAMO: Laboratory of Morphology and Organogenesis
NC+: New Cases
PCR-SSP: Polymerase Chain Reaction With Sequence-Specific Primers
SARS-CoV-2: Severe Acute Respiratory Syndrome Coronavirus 2
SC+: Severe Cases
TMPRSS2: Transmembrane Serine Protease 2

## References

[1] Zhu N, Zhang D, Wang W, Li X, Yang B, Song J (2020). A novel coronavirus from patients with Pneumonia in China, 2019. N Engl J Med.

[2] WHO. WHO Director-General’s opening remarks at the media briefing on COVID-19–11. March 2020. 2020. https://www.who.int/director-general/speeches/detail/who-director-general-s-opening-remarks-at-the-media-briefing-on-covid-19---11-march-2020. Accessed 25 Jan 2023.

[3] WHO. WHO Coronavirus (COVID-19) Dashboard. 2023. https://covid19.who.int.

[4] Kagoné TS, Bisseye C, Méda N, Testa J, Pietra V, Kania D (2014). A variant of DC-SIGN gene promoter associated with resistance to HIV-1 in serodiscordant couples in Burkina Faso. Asian Pac J Trop Med.

[5] Compaore TR, Soubeiga ST, Ouattara AK, Obiri-Yeboah D, Tchelougou D, Maiga M (2016). APOBEC3G variants and Protection against HIV-1 infection in Burkina Faso. PLoS ONE.

[6] Wang B, Huang G, Wang D, Li A, Xu Z, Dong R (2010). Null genotypes of GSTM1 and GSTT1 contribute to hepatocellular carcinoma risk: evidence from an updated meta-analysis. J Hepatol.

[7] Kokkotou E, Philippon V, Guèye-Ndiaye A, Mboup S, Wang WK, Essex M (1998). Role of the CCR5 delta 32 allele in resistance to HIV-1 infection in west Africa. J Hum Virol.

[8] Sma H, M T, Sy H, Mr AT. P, J S: Human gene polymorphisms and their possible impact on the clinical outcome of SARS-CoV-2 infection. Arch Virol 2021; 1668. 10.1007/s00705-021-05070-6 PMC8088757 33934196

[9] Ellinghaus D, Degenhardt F, Bujanda L, Buti M, Albillos A, Invernizzi P, et al. Genomewide Association study of severe Covid-19 with respiratory failure. N Engl J Med. 2020. NEJMoa2020283. 10.1056/NEJMoa2020283 PMC7315890 32558485

[10] Klein J, Sato A (2000). The HLA System. N Engl J Med.

[11] Wei L-z, Wang H-l, Liu X, Lu Y-p, Xu F, Yuan J-q (2014). Meta-analysis on the relationship between HLA-DRBl Gene Polymorphism and Cervical Cancer in Chinese Population. PLoS ONE.

[12] Tomić S, Đokić J, Stevanović D, Ilić N, Gruden-Movsesijan A, Dinić M (2021). Reduced expression of autophagy markers and expansion of myeloid-derived suppressor cells correlate with poor T cell response in severe COVID-19 patients. Front Immunol.

[13] Dobrijević Z, Gligorijević N, Šunderić M, Penezić A, Miljuš G, Tomić S et al. The association of human leucocyte antigen (HLA) alleles with COVID-19 severity: a systematic review and meta‐analysis. Rev Med Virol 2022:e2378. 10.1002/rmv.2378 PMC9349710 35818892

[14] Ouattara AK, Traoré L, Compaoré TR, Zohoncon TM, Simporé J (2023). G6PD Deficiency and COVID-19 in Burkina Faso: a possible link?. J Biosci Med.

[15] Klein S, Pekosz A, Park HS, Ursin R, Shapiro J, Benner S et al. Sex, age, and hospitalization drive antibody responses in a COVID-19 convalescent plasma donor population. *medRxiv* 2020. 10.1172/JCI142004 PMC7598041 32764200

[16] Scully EP, Haverfield J, Ursin RL, Tannenbaum C, Klein SL (2020). Considering how biological sex impacts immune responses and COVID-19 outcomes. Nat Rev Immunol.

[17] Taslem Mourosi J, Anwar S, Hosen MJ (2022). The sex and gender dimensions of COVID-19: a narrative review of the potential underlying factors. Infect Genet Evol.

[18] Fadl N, Ali E, Salem TZ (2021). COVID-19: risk factors Associated with Infectivity and Severity. Scand J Immunol.

[19] Akbar AN, Gilroy DW (2020). Aging immunity may exacerbate COVID-19. Science.

[20] Chen Y, Klein SL, Garibaldi BT, Li H, Wu C, Osevala NM (2021). Aging in COVID-19: vulnerability, immunity and intervention. Ageing Res Rev.

[21] Zouré AA, Amegnona LJ, Zongo N, Kiendrebeogo IT, Sorgho PA, Zongo FI (2021). Carriage of HLA-DRB1*11 and 1*12 alleles and risk factors in patients with breast cancer in Burkina Faso. Open Life Sci.

[22] Lallogo TD, Djigma FW, Sorgho PA, Martinson JJ, Compaore TR, Traore L (2022). KIR2DL5B and HLA DRB1*12 alleles seems to be associated with protection against HIV-1 in serodiscordant couples in Burkina Faso. J Med Virol.

[23] Jain SK, Parsanathan R, Levine SN, Bocchini JA, Holick MF, Vanchiere JA (2020). The potential link between inherited G6PD deficiency, oxidative stress, and vitamin D deficiency and the racial inequities in mortality associated with COVID-19. Free Radic Biol Med.

[24] Poulton K, Wright P, Hughes P, Savic S, Welberry Smith M, Guiver M (2020). A role for human leucocyte antigens in the susceptibility to SARS-Cov‐2 infection observed in transplant patients. Int J Immunogenet.

[25] Matsuura H, Fujii S, Matsui Y, Sugiura Y, Akiyama H, Miura Y (2022). An association between a positive direct antiglobulin test and HLA-DR12 in COVID-19. Ann Hematol.

[26] Migliorini F, Torsiello E, Spiezia F, Oliva F, Tingart M, Maffulli N (2021). Association between HLA genotypes and COVID-19 susceptibility, severity and progression: a comprehensive review of the literature. Eur J Med Res.

[27] Vigón L, Galán M, Torres M, Martín-Galiano AJ, Rodríguez-Mora S, Mateos E (2022). Association between HLA-C alleles and COVID-19 severity in a pilot study with a spanish Mediterranean caucasian cohort. PLoS ONE.

[28] Naemi FMA, Al-adwani S, Al-khatabi H, Al-nazawi A (2021). Frequency of HLA alleles among COVID-19 infected patients: preliminary data from Saudi Arabia. Virology.

[29] Dobrijević Z, Gligorijević N, Šunderić M, Penezić A, Miljuš G, Tomić S (2023). The association of human leucocyte antigen (HLA) alleles with COVID-19 severity: a systematic review and meta-analysis. Rev Med Virol.

[30] Ertosun MG, Özkan Ö, Darbaş Ş, Özel D, BİLGE U, Sayin Ekinci N (2022). The relationship between COVID-19 and HLA in kidney transplant recipients, an evaluation of predictive and prognostic factors. Clin Transplant.

[31] Yasuda S, Suzuki S, Yanagisawa S, Morita H, Haisa A, Satomura A (2023). HLA typing of patients who developed subacute thyroiditis and Graves’ disease after SARS-CoV-2 vaccination: a case report. BMC Endocr Disord.

[32] Wang W, Zhang W, Zhang J, He J, Zhu F (2020). Distribution of HLA allele frequencies in 82 chinese individuals with coronavirus disease-2019 (COVID‐19). HLA.

[33] Wang W, Zhang W, Zhang J, He J, Zhu F: Distribution of HLA allele frequencies in 82 Chinese individuals with coronavirus disease-2019 (COVID‐19). *HLA* 2020; 962:194–196. 10.1111/tan.13941 PMC7276866 32424945

