# Supplementary material for: Association of HLA-DRB1*11 and HLA-DRB1*12 gene polymorphism with COVID-19 in Burkina Faso
Source: BMC Med Genomics. 2023 Oct 16;16:246. doi: 10.1186/s12920-023-01684-8 (PMC10577973; doi:10.1186/s12920-023-01684-8)
Supplement: Supplementary file 1 — Supplementary Material 1 [file 12920_2023_1684_MOESM1_ESM.docx]

**Supplementary file:** PCR-SSP detection of *HLA-DRB1*11* and *HLA-*
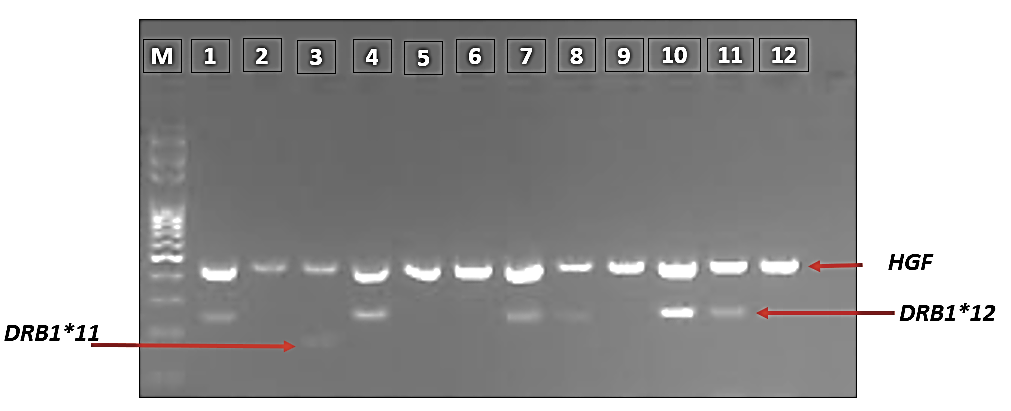
*DRB1*12* alleles

**Image 1:** PCR-SSP detection of *HLA-DRB1*11* and *HLA- DRB1*12* alleles





**Image 2:** PCR-SSP detection of *HLA-DRB1*11* and *HLA- DRB1*12* alleles





**Image 3:** PCR-SSP detection of *HLA-DRB1*11* and *HLA- DRB1*12* alleles


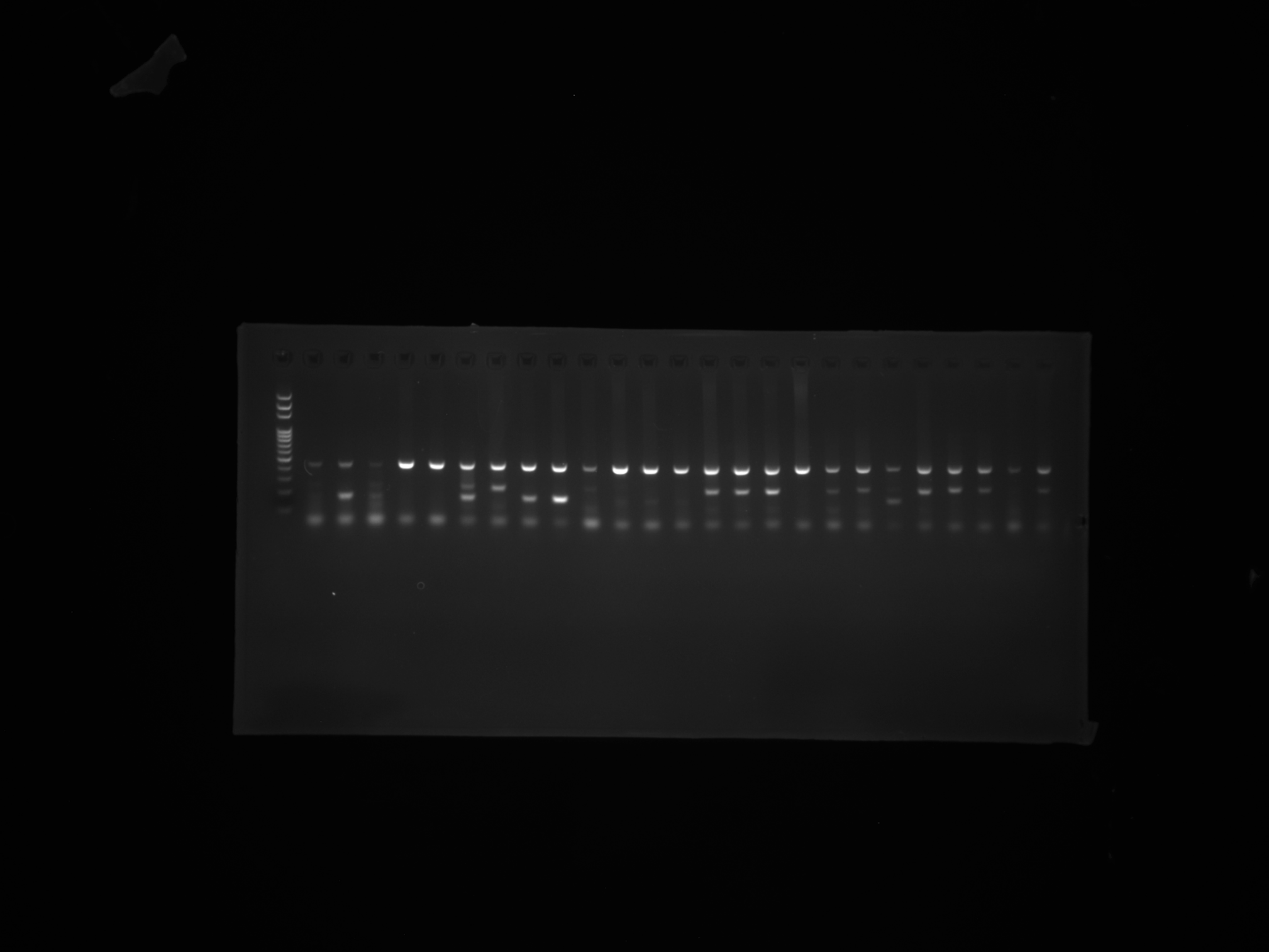


**Image 4:** PCR-SSP detection of *HLA-DRB1*11* and *HLA- DRB1*12* alleles


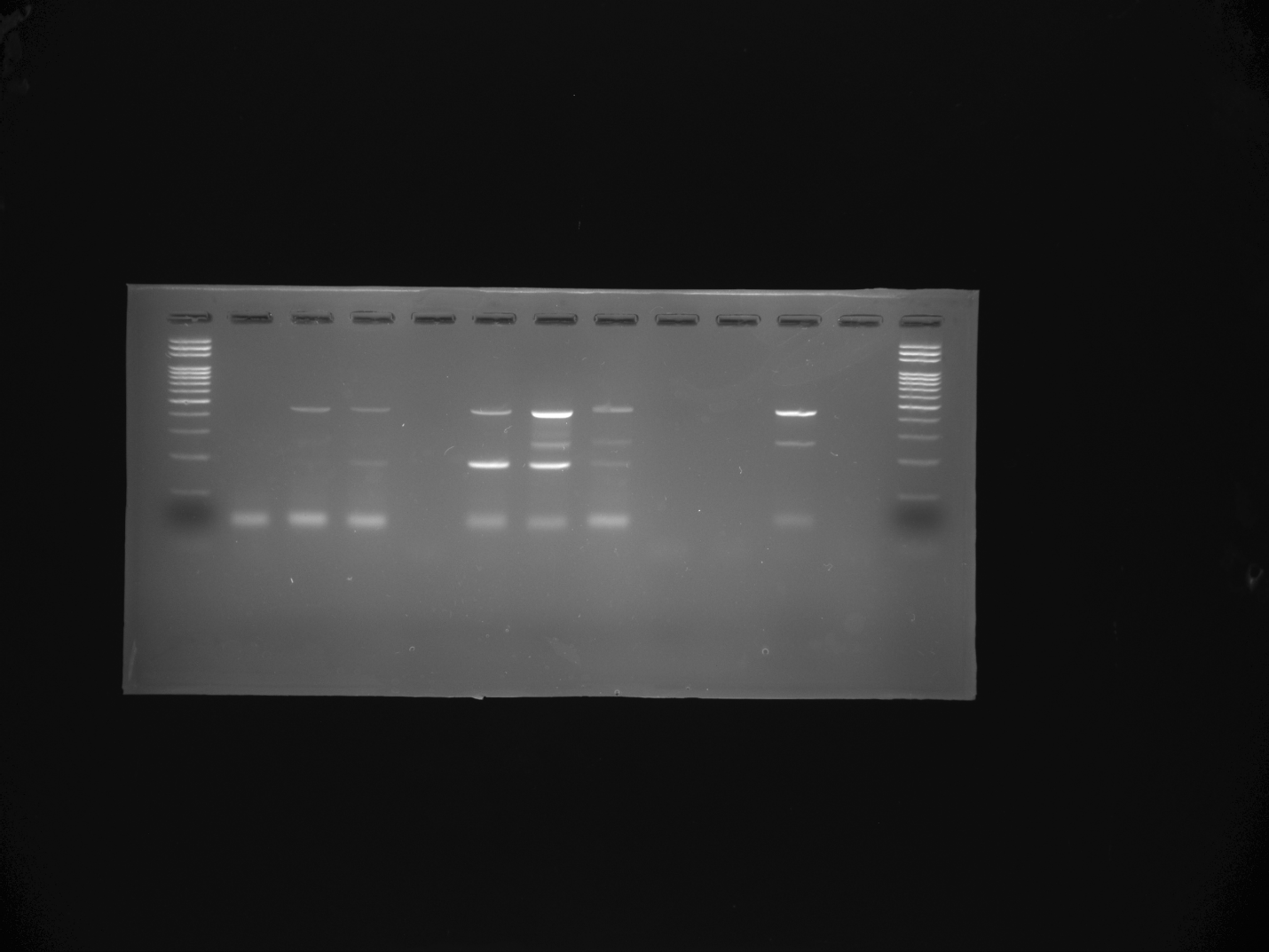


**Image 5:** PCR-SSP detection of *HLA-DRB1*11* and *HLA- DRB1*12* alleles
